# Tubo-Ovarian Abscess Formation in Users of Intrauterine Devices Remote From Insertion: A Report of Three Cases

**DOI:** 10.1155/S106474499600018X

**Published:** 1996

**Authors:** Marc R. Toglia, Joseph I. Schaffer

**Affiliations:** 1 Department of Obstetrics, Gynecology, and Reproductive Medicine State University of New York at Stony Brook Stony Brook NY USA; 2 Vanguard Graduate Gynecology Associates 1740 South Street Suite 306 Philadelphia PA 19146 USA

## Abstract

*Background:* The association between tubo-ovarian abscess formation and the presence of an intrauterine device (IUD) is well recognized. It has been suggested that the risk of upper-genital-tract infection is highest during the immediate period following the insertion of an IUD, returning to baseline by 5 months postinsertion. We present 3 cases of women who, 10–21 years after insertion of their IUDs, developed tubo-ovarian abscesses that were not causally related to sexually transmitted diseases (STDs) or actinomycetes.

*Cases:* Three women, ages 39–47 years, presented to our gynecology service for evaluation of abdominal pain. One woman had bilateral tubo-ovarian abscesses and the other 2 had unilateral tubo-ovarian abscesses. All 3 were IUD users, with an interval from IUD insertion to presentation of 10–21 years. In each case, the cervical cultures for gonorrhea and chlamydia were negative at presentation and the sexual history was not consistent with an STD mode of spread. All 3 women initially received broad-spectrum antibiotics, but 2 eventually required definitive surgical therapy.

*Conclusion:* Long-term users of IUDs remain at risk for serious, indolent pelvic infections. These women should be counseled by their gynecologists on an ongoing basis as to this persistent risk. Tubo-ovarian abscess should be strongly considered in the differential diagnosis of an IUD user who presents with an adnexal mass, fever, or abdominal pain.


Infectious Diseases in Obstetrics and Gynecology 4:85–88 (1996)
© 1996 Wiley-Liss, Inc.

### Tubo-Ovarian Abscess Formation in Users of Intrauterine Devices Remote From Insertion: A Report of Three Cases

**Marc R. Toglia and Joseph I. Schaffer**

*Department of Obstetrics, Gynecology, and Reproductive Medicine, State University of New York at Stony Brook, Stony Brook, NY*

ABSTRACT

***Background:*** **The association between tubo-ovarian abscess formation and the presence of an intrauterine device (IUD) is well recognized. It has been suggested that the risk of upper-genital-tract infection is highest during the immediate period following the insertion of an IUD, returning to baseline by 5 months postinsertion. We present 3 cases of women who, 10–21 years after insertion of their IUDs, developed tubo-ovarian abscesses that were not causally related to sexually transmitted diseases (STDs) or actinomycetes.**

***Cases:*** **Three women, ages 39–47 years, presented to our gynecology service for evaluation of abdominal pain. One woman had bilateral tubo-ovarian abscesses and the other 2 had unilateral tubo-ovarian abscesses. All 3 were IUD users, with an interval from IUD insertion to presentation of 10–21 years. In each case, the cervical cultures for gonorrhea and chlamydia were negative at presentation and the sexual history was not consistent with an STD mode of spread. All 3 women initially received broad-spectrum antibiotics, but 2 eventually required definitive surgical therapy.**

***Conclusion:*** **Long-term users of IUDs remain at risk for serious, indolent pelvic infections. These women should be counseled by their gynecologists on an ongoing basis as to this persistent risk. Tubo-ovarian abscess should be strongly considered in the differential diagnosis of an IUD user who presents with an adnexal mass, fever, or abdominal pain.** © 1996 Wiley-Liss, Inc.

KEY WORDS

Pelvic inflammatory disease, pelvic abscess, salpingitis

Tubo-ovarian abscess formation is an uncommon but serious complication associated with the use of an intrauterine device (IUD).[1–3] Recent studies have suggested that the risk of a pelvic infection is greatest during the first several months of IUD use, with the risk returning to baseline at about 5 months postinsertion.[4–6] An upper-genital-tract infection occurring after this period is felt to be related to sexually transmitted diseases (STDs) or actinomycetes.[7,8]

During the last 17 months, we have managed 3 patients with tubo-ovarian abscess formation that developed while an IUD was present. In each case, the abscess formation, occurring at a time remote from the insertion of the IUD, could not be causally related to an identifiable STD or actinomycetes. In addition, each patient's history of sexual activity made it unlikely that an STD was the etiology for the abscess formation. Cervical cultures for gonorrhea and a DNA probe for chlamydia were performed at the initial presentation of each patient. At the time of removal, each IUD was cultured for both aerobic and anaerobic organisms, including actinomycetes. The anaerobic cultures were processed in thioglycolate broth, CDC blood agar, and CDC blood agar Phenol ethyl alcohol media (PEA)

Address correspondence/reprint requests to Dr. Marc R. Toglia, Vanguard Graduate Gynecology Associates, 1740 South Street, Suite 306, Philadelphia, PA 19146.

Received March 25, 1996
Accepted June 11, 1996

for a minimum of 14 days. Portions of the abscess walls were similarly cultured for both aerobic and anaerobic organisms by a microbiology laboratory. The pathology laboratory was requested to identify actinomycetes in the specimens by histologic and special staining techniques.

### CASE REPORTS

### Case 1

A 43-year-old white female, $G_1P_1$, presented to our gynecology clinic on September 27, 1994, with a 2-day history of lower abdominal pain and heavy vaginal bleeding. The patient admitted to anorexia, diarrhea, and chills but denied any fever. She had had a Safe-T Coil in place since 1973. She was involved in a monogamous relationship with her spouse. Her primary physician had ordered a pelvic ultrasound which revealed a complex left adnexal mass, 9.2 × 6.2 × 8.2 cm.

On presentation, she was noted to be afebrile, with a temperature of 37°C. Her pulse rate was 72 and her blood pressure was 105/64. Her clinical examination was remarkable for mild tenderness in the lower abdomen but normal active bowel sounds and no palpable masses. The pelvic examination revealed the string of the IUD but no mucopurulent discharge. On her bimanual examination, an 8-cm cystic, tender mass in the cul-de-sac and bilateral adnexal tenderness were noted. Cervical motion tenderness was absent. A rectal examination confirmed the above findings, and her stool was guaiac negative. Her WBC count was 10,800, the erythrocyte sedimentation rate was 98, and a CA125 was 21.5. Cervical cultures for gonorrhea and chlamydia were negative.

The patient underwent an exploratory laparotomy on October 3, 1994. After induction of anesthesia, the IUD was removed. The operative findings revealed an 8-cm left tubo-ovarian abscess, right hydrosalpinx, and severe pelvic adhesions. She underwent a total abdominal hysterectomy and bilateral salpingo-oophorectomy. Intraoperative spill of the abscess cavity occurred, so the pelvis was copiously irrigated. Postoperatively, she was treated with intravenous (IV) cefoxitin and doxycycline for 5 days, and her wound was closed by delayed primary intention prior to her discharge from the hospital. The cultures of the abscess revealed *Prevotella bivius* and *Fusobacterium*. The pathology revealed chronic endometritis and a left tubo-ovarian abscess.

### Case 2

A 39-year-old nulligravid white female presented through the emergency room on December 28, 1994, with a 10-day history of fever and lower abdominal pain. She had been seen at the onset of her illness at an urgent-care center and treated for gastroenteritis. The patient reported that she was not sexually active, with her last act of coitus having been 4 months prior. Ten years earlier in Norway, she had had a Copper 7 IUD inserted.

On her presentation, her temperature was 38.6°C, pulse rate was 93, and blood pressure was 121/59. Her examination was remarkable for diminished bowel sounds in all 4 quadrants, mild abdominal distention, and tenderness without rebound in both lower quadrants. Her pelvic examination revealed the IUD string but no gross purulent discharge. A bimanual examination revealed marked cervical motion tenderness and left adnexal tenderness with a palpable fullness. Her rectal examination was remarkable for the stool's being guaiac positive. On her admission, the WBC count was 22,700 and the erythrocyte sedimentation rate was 32. Cervical cultures for gonorrhea and chlamydia were negative.

The patient was admitted and started on IV cefoxitin and doxycycline. A pelvic ultrasound revealed a left tubo-ovarian complex, 3.6 × 4.2 × 5.2 cm, and a normal right adnexa. As her ileus worsened, she was given nothing by mouth. The patient remained febrile. On her third hospital day, a computerized tomographic scan confirmed a left tubo-ovarian abscess but no other abdominal or pelvic pathology. Because of the prolonged ileus, a general surgery consultation was obtained. Their service agreed that a gastrointestinal etiology such as an appendiceal or diverticular abscess was not likely to be the cause of her illness. Her antibiotic regimen was changed to ampicillin, gentamicin, and metronidazole. The patient defervesced and her ileus resolved in the ensuing 3 days. She was discharged from the hospital on day 7 after remaining afebrile for 72 h. She was seen in the outpatient gynecology clinic at 2 and 4 weeks after her discharge. Her clinical examinations on those visits confirmed resolution of the left-sided adnexal mass.

### Case 3

A 47-year-old white female, $G_3P_2A_1$, presented to the emergency room on February 10, 1996, with a 1-day history of fever to 38.4°C and a pinkish vaginal discharge. She had had a Lippes Loop (Ortho Pharmaceutical, Raritan, NJ) inserted 20 years earlier. She denied being sexually active for the previous 12 years.

Her clinical examination included a temperature of 38.2°C, pulse rate of 90, and blood pressure of 122/70. An abdominal examination revealed minimal tenderness with no rebound to deep palpation in both lower quadrants. The pelvic examination revealed a clear but malodorous discharge and the IUD string. The bimanual examination was significant for bilateral adnexal and cervical motion tenderness. The laboratory studies revealed a WBC count of 15,000. A pelvic ultrasound showed an ill-defined fluid collection, 3.3 × 4.8 cm, surrounding a normal-appearing left ovary. Cervical cultures for gonorrhea and chlamydia were negative.

The patient was initially managed for pelvic inflammatory disease with IV cefoxitin and oral doxycycline, and the IUD was removed. Her maximum temperature was 40.3°C occurring on the second hospital day. By the third hospital day, her clinical examination revealed minimal improvement, but she remained febrile. Her WBC count remained at 16,600. Her antibiotics were changed to ticarcillin/clavulanate and gentamicin. She remained febrile despite the change in antibiotics. On her seventh hospital day, a computerized tomographic scan of the pelvis revealed a complex cystic mass, 7.5 × 5.8 × 7.0 cm, in the left adnexa consistent with a tubo-ovarian abscess. Given the lack of clinical improvement with broad-spectrum antibiotics, we decided to perform a laparotomy on the following day.

At the laparotomy, bilateral tubo-ovarian abscesses, 10 cm on the left and 4 cm on the right, were discovered. A total abdominal hysterectomy, bilateral salpingo-oophorectomy, adhesiolysis, and left ureterolysis were performed. A Jackson-Pratt drain was placed over the closed vaginal cuff. On the third postoperative day, the drain broke off during removal. She was taken back to the operating room for the removal of a single fascial suture that was discovered to be attached to the drain. She was discharged home 2 days later.

Cultures of the endometrial cavity from her admission and later from the abscess grew *Bacteroides fragilis* and *Lactobacillus jensenii*. The pathology revealed bilateral tubo-ovarian abscesses and adenomyosis.

### DISCUSSION

These 3 cases illustrate that a woman using an IUD remains at risk for the development of pelvic abscess formation for as long as the IUD is present. In our case series, the ages ranged from 39 to 47 years and the duration of IUD use ranged from 10 to 21 years prior to the clinical presentation. Two of the 3 women were not sexually active at the time of diagnosis, and cervical cultures at the time of presentation did not implicate common STDs such as gonorrhea or chlamydia. None of these women showed evidence of immunocompromise or any other debilitating or chronic medical condition that may have predisposed them to such an unusual infection.

Although the route and mode of infection remain unclear in each of these patients, it is likely that an ascending infection from the vaginal flora occurred. The IUD is known to disrupt the normal protective mechanism of the endometrial cavity which may continually promote the possibility of infection. It has also been proposed that the IUD tail allows easy access of the vaginal bacteria to the upper genital tract.[9]

We conclude that long-term IUD users remain at risk for serious, indolent pelvic infections and that clinicians should continuously address this issue with these patients during each encounter. Two of the 3 women in this study required definitive surgical therapy for their disease despite aggressive and prolonged antibiotic regimens. A tubo-ovarian abscess should be considered high in the differential diagnosis in any IUD user who presents with a tubo-ovarian complex, fever, or abdominal pain. We suggest that all IUDs be removed at a time when contraception is no longer an issue for the patient, e.g., a woman in the perimenopausal years.

### REFERENCES

1. Taylor ES, McMillan JH, Greer BE, Droegemueller W, Thompson HE: The intrauterine device and tubo-ovarian abscess. Am J Obstet Gynecol 123:338–341, 1975.
2. Dawood MY, Birnbaum SJ: Unilateral tubo-ovarian abscess and intrauterine contraceptive device. Obstet Gynecol 46:429–432, 1975.

3. Duckman S, Suarez J, Tantakesem P: Tubo-ovarian abscess and the intrauterine device. Am J Obstet Gynecol 115:1157–1158, 1975.
4. Lee NC, Rubin GL, Borucki R: The intrauterine device and pelvic inflammatory disease revisited: New results from the Women's Health Study. Obstet Gynecol 72:1–6, 1988.
5. Kessel E: Pelvic inflammatory disease with intrauterine device use: A reassessment. Fertil Steril 51:1–11, 1989.
6. Grimes DA: Intrauterine devices and pelvic inflammatory disease: Recent developments. Contraception 36:97–109, 1987.
7. Pearlman M, Frantz AC, Floyd WS, Faro S: Abdominal wall actinomyces associated with an intrauterine device: A case report. J Reprod Med 36:398–402, 1991.
8. Purdie DW, Carty MJ, McLeod TI: Tubo-ovarian actinomycosis and IUCD. Br Med J 2:1392, 1977.
9. Sparks RA, Purrier BGA, Watt PJ, Ectein M: Bacterial colonization of uterine cavity: Role of tailed intrauterine contraceptive devices. Br Med J 282:1189–1191, 1981.
